# Supplementary material for: Unveiling the Bioactive Potential of the Invasive Jellyfish Phyllorhiza punctata Through Integrative Transcriptomic and Proteomic Analyses
Source: Biomolecules. 2025 Aug 4;15(8):1121. doi: 10.3390/biom15081121 (PMC12383608; doi:10.3390/biom15081121)
Supplement: Supplementary file 1 [file biomolecules-15-01121-s001.zip › Suplementary Material/FileS3/PEPFOLD4/XP_065054309_3679/clusters.html]

 PEP-FOLD clusters


Download archive of all models  here   

|  |  |  |
| --- | --- | --- |
| # | file | sOPEP |
| # cluster 1 - Size: 11 |  |  |
|  | XP\_065054309\_3679-model1 | -26.3771 |
|  | XP\_065054309\_3679-model1.1 | -23.4044 |
|  | XP\_065054309\_3679-model1.2 | -22.8644 |
|  | XP\_065054309\_3679-model1.3 | -22.5732 |
|  | XP\_065054309\_3679-model1.4 | -22.4296 |
|  | XP\_065054309\_3679-model1.5 | -21.988 |
|  | XP\_065054309\_3679-model1.6 | -21.9439 |
|  | XP\_065054309\_3679-model1.7 | -20.6099 |
|  | XP\_065054309\_3679-model1.8 | -19.6823 |
|  | XP\_065054309\_3679-model1.9 | -19.3635 |
|  | XP\_065054309\_3679-model1.10 | -19.0136 |
| # cluster 2 - Size: 3 |  |  |
|  | XP\_065054309\_3679-model2 | -25.2655 |
|  | XP\_065054309\_3679-model2.1 | -21.7442 |
|  | XP\_065054309\_3679-model2.2 | -19.3374 |
| # cluster 3 - Size: 15 |  |  |
|  | XP\_065054309\_3679-model3 | -24.5998 |
|  | XP\_065054309\_3679-model3.1 | -24.4302 |
|  | XP\_065054309\_3679-model3.2 | -24.3754 |
|  | XP\_065054309\_3679-model3.3 | -23.9162 |
|  | XP\_065054309\_3679-model3.4 | -23.3398 |
|  | XP\_065054309\_3679-model3.5 | -22.8777 |
|  | XP\_065054309\_3679-model3.6 | -22.1141 |
|  | XP\_065054309\_3679-model3.7 | -22.0212 |
|  | XP\_065054309\_3679-model3.8 | -18.2743 |
|  | XP\_065054309\_3679-model3.9 | -18.0838 |
|  | XP\_065054309\_3679-model3.10 | -17.9566 |
|  | XP\_065054309\_3679-model3.11 | -17.6293 |
|  | XP\_065054309\_3679-model3.12 | -17.4052 |
|  | XP\_065054309\_3679-model3.13 | -15.4429 |
|  | XP\_065054309\_3679-model3.14 | -11.7766 |
| # cluster 4 - Size: 2 |  |  |
|  | XP\_065054309\_3679-model4 | -24.5779 |
|  | XP\_065054309\_3679-model4.1 | -22.1591 |
| # cluster 5 - Size: 8 |  |  |
|  | XP\_065054309\_3679-model5 | -24.0951 |
|  | XP\_065054309\_3679-model5.1 | -23.8627 |
|  | XP\_065054309\_3679-model5.2 | -23.6354 |
|  | XP\_065054309\_3679-model5.3 | -20.8528 |
|  | XP\_065054309\_3679-model5.4 | -19.8337 |
|  | XP\_065054309\_3679-model5.5 | -19.7874 |
|  | XP\_065054309\_3679-model5.6 | -18.5751 |
|  | XP\_065054309\_3679-model5.7 | -15.3388 |
| # cluster 6 - Size: 3 |  |  |
|  | XP\_065054309\_3679-model6 | -23.6521 |
|  | XP\_065054309\_3679-model6.1 | -17.9575 |
|  | XP\_065054309\_3679-model6.2 | -14.8702 |
| # cluster 7 - Size: 1 |  |  |
|  | XP\_065054309\_3679-model7 | -23.6134 |
| # cluster 8 - Size: 3 |  |  |
|  | XP\_065054309\_3679-model8 | -21.8611 |
|  | XP\_065054309\_3679-model8.1 | -19.9649 |
|  | XP\_065054309\_3679-model8.2 | -15.7751 |
| # cluster 9 - Size: 1 |  |  |
|  | XP\_065054309\_3679-model9 | -21.2216 |
| # cluster 10 - Size: 3 |  |  |
|  | XP\_065054309\_3679-model10 | -20.6725 |
|  | XP\_065054309\_3679-model10.1 | -18.1082 |
|  | XP\_065054309\_3679-model10.2 | -15.0577 |
| # cluster 11 - Size: 1 |  |  |
|  | XP\_065054309\_3679-model11 | -19.1507 |
| # cluster 12 - Size: 1 |  |  |
|  | XP\_065054309\_3679-model12 | -19.1345 |
| # cluster 13 - Size: 1 |  |  |
|  | XP\_065054309\_3679-model13 | -18.6564 |
| # cluster 14 - Size: 1 |  |  |
|  | XP\_065054309\_3679-model14 | -16.7179 |
| # cluster 15 - Size: 2 |  |  |
|  | XP\_065054309\_3679-model15 | -15.6492 |
|  | XP\_065054309\_3679-model15.1 | -11.2551 |
| # cluster 16 - Size: 1 |  |  |
|  | XP\_065054309\_3679-model16 | -14.2492 |
| # cluster 17 - Size: 4 |  |  |
|  | XP\_065054309\_3679-model17 | -13.7689 |
|  | XP\_065054309\_3679-model17.1 | -11.7471 |
|  | XP\_065054309\_3679-model17.2 | -10.8016 |
|  | XP\_065054309\_3679-model17.3 | -10.2577 |
| # cluster 18 - Size: 1 |  |  |
|  | XP\_065054309\_3679-model18 | -13.7187 |
| # cluster 19 - Size: 5 |  |  |
|  | XP\_065054309\_3679-model19 | -13.3913 |
|  | XP\_065054309\_3679-model19.1 | -12.0414 |
|  | XP\_065054309\_3679-model19.2 | -11.3151 |
|  | XP\_065054309\_3679-model19.3 | -10.9626 |
|  | XP\_065054309\_3679-model19.4 | -10.9524 |
| # cluster 20 - Size: 3 |  |  |
|  | XP\_065054309\_3679-model20 | -13.3892 |
|  | XP\_065054309\_3679-model20.1 | -10.4372 |
|  | XP\_065054309\_3679-model20.2 | -10.3056 |
| # cluster 21 - Size: 2 |  |  |
|  | XP\_065054309\_3679-model21 | -12.6082 |
|  | XP\_065054309\_3679-model21.1 | -10.552 |
| # cluster 22 - Size: 3 |  |  |
|  | XP\_065054309\_3679-model22 | -12.4975 |
|  | XP\_065054309\_3679-model22.1 | -12.0755 |
|  | XP\_065054309\_3679-model22.2 | -11.8033 |
| # cluster 23 - Size: 1 |  |  |
|  | XP\_065054309\_3679-model23 | -12.348 |
| # cluster 24 - Size: 3 |  |  |
|  | XP\_065054309\_3679-model24 | -12.2673 |
|  | XP\_065054309\_3679-model24.1 | -11.7007 |
|  | XP\_065054309\_3679-model24.2 | -11.3746 |
| # cluster 25 - Size: 3 |  |  |
|  | XP\_065054309\_3679-model25 | -12.1197 |
|  | XP\_065054309\_3679-model25.1 | -10.8019 |
|  | XP\_065054309\_3679-model25.2 | -10.7204 |
| # cluster 26 - Size: 1 |  |  |
|  | XP\_065054309\_3679-model26 | -11.7901 |
| # cluster 27 - Size: 4 |  |  |
|  | XP\_065054309\_3679-model27 | -11.6356 |
|  | XP\_065054309\_3679-model27.1 | -11.4028 |
|  | XP\_065054309\_3679-model27.2 | -11.2146 |
|  | XP\_065054309\_3679-model27.3 | -10.3577 |
| # cluster 28 - Size: 1 |  |  |
|  | XP\_065054309\_3679-model28 | -11.5898 |
| # cluster 29 - Size: 3 |  |  |
|  | XP\_065054309\_3679-model29 | -11.4385 |
|  | XP\_065054309\_3679-model29.1 | -10.8188 |
|  | XP\_065054309\_3679-model29.2 | -10.6906 |
| # cluster 30 - Size: 3 |  |  |
|  | XP\_065054309\_3679-model30 | -11.3952 |
|  | XP\_065054309\_3679-model30.1 | -11.0416 |
|  | XP\_065054309\_3679-model30.2 | -10.4984 |
| # cluster 31 - Size: 1 |  |  |
|  | XP\_065054309\_3679-model31 | -11.2283 |
| # cluster 32 - Size: 1 |  |  |
|  | XP\_065054309\_3679-model32 | -11.0048 |
| # cluster 33 - Size: 1 |  |  |
|  | XP\_065054309\_3679-model33 | -10.9645 |
| # cluster 34 - Size: 1 |  |  |
|  | XP\_065054309\_3679-model34 | -10.8454 |
| # cluster 35 - Size: 1 |  |  |
|  | XP\_065054309\_3679-model35 | -10.4949 |
| # cluster 36 - Size: 1 |  |  |
|  | XP\_065054309\_3679-model36 | -10.2718 |
|  |  |  |
